# Advantages and Applications of Total-Body PET Scanning

**DOI:** 10.3390/diagnostics12020426

**Published:** 2022-02-07

**Authors:** Sanaz Katal, Liesl S. Eibschutz, Babak Saboury, Ali Gholamrezanezhad, Abass Alavi

**Affiliations:** 1 Independent Researcher, Melbourne 3000, Australia; sanaz.katal@gmail.com; 2 Department of Radiology, Keck School of Medicine, University of Southern California (USC), Los Angeles, CA 90007, USA; liesl.eibschutz@med.usc.edu (L.S.E.); ali.gholamrezanezhad@med.usc.edu (A.G.); 3 Department of Radiology and Imaging Sciences, Clinical Center, National Institutes of Health (NIH), Bethesda, MD 20892, USA; babak.saboury@nih.gov; 4 Department of Radiology, Hospital of the University of Pennsylvania, Philadelphia, PA 19104, USA

**Keywords:** total-body PET, oncology, FDG, drug development, PET/CT, inflammatory disease

## Abstract

Recent studies have focused on the development of total-body PET scanning in a variety of fields such as clinical oncology, cardiology, personalized medicine, drug development and toxicology, and inflammatory/infectious disease. Given its ultrahigh detection sensitivity, enhanced temporal resolution, and long scan range (1940 mm), total-body PET scanning can not only image faster than traditional techniques with less administered radioactivity but also perform total-body dynamic acquisition at a longer delayed time point. These unique characteristics create several opportunities to improve image quality and can provide a deeper understanding regarding disease detection, diagnosis, staging/restaging, response to treatment, and prognostication. By reviewing the advantages of total-body PET scanning and discussing the potential clinical applications for this innovative technology, we can address specific issues encountered in routine clinical practice and ultimately improve patient care.

## 1. Introduction

Over the past few decades, researchers around the world have been intent on improving the sensitivity and resolution of positron emission tomography (PET) imaging. Recent modifications include the introduction of novel gating methods, iterative reconstruction algorithms, detectors with optimized geometry, time-of-flight technologies, and integration of computed tomography (CT) imaging. While these changes have remarkably improved PET quality, the limited axial coverage of current PET scanners continues to be a significant constraint in the face of current imaging systems. With an axial coverage of 15–30 cm, PET detectors ultimately collect a limited number of coincidence photons, thus generating images deemed too noisy given low counting statistics. Moreover, the limited temporal resolution associated with PET systems makes obtaining dynamic imaging studies using tracer kinetic modeling methods a challenging task.

To overcome these limitations, total-body-length PET scanners were developed, thus extending the axial field of view (AFOW) to cover the entire human body. In addition, by increasing the number of detectors, most emitted photons can be captured, thus dramatically increasing sensitivity, and allowing for simultaneous dynamic acquisition from all tissues of interest. Ultimately, this ultrasensitive high-performance device offers many advantages over conventional systems, such as an enhanced signal-to-noise ratio (SNR), image quality with a shorter acquisition time, and lesser injected radioactivity.

Recently, healthcare agencies have recognized the utility of total-body PET scanning and proposed a variety of total body scanners for clinical and research purposes (Table 1). This piece will analyze the clinical advantages of total-body PET/CT and discuss the practical implications and conditions in which the scanner could be applied in the future. 

## 2. Major Advantages of Total-Body PET Scanners

### 2.1. Increased Signal-to-Noise Ratio

While the radiation exposure associated with PET/CT imaging often presents as a major concern, many characteristics associated with the total-body scanner can ease the radiation burden. For instance, the ultrahigh sensitivity offered by the total-body scanner allows it to provide comparable images with remarkably lower activity. This can be attributed to the signal-to-noise ratio, which is directly correlated with the sensitivity of the scanner, acquisition time, and activity administered. The SNR value also determines the image quality, with a higher SNR indicating a higher-quality image. Thus, total-body scanning’s high sensitivity (up to 68 times higher than PET/CT), even in the setting of lower administered activity, will still yield an increased SNR value and allow for a 40-fold reduction in radioactivity dose [2]. Lower activity also reduces dead-time count loss, thus further improving image quality [3]. Ultimately, lower administered activity can enable previously vulnerable populations such as young children and pregnant women to utilize PET/CT technology. Moreover, adults requiring longitudinal studies may be able to undergo repeated scans.

### 2.2. Reduction in Imaging Time

One of the primary benefits associated with total-body scanning is the reduction in imaging time. Certain authors have estimated that this imaging technique decreases length in imaging by a factor of 24 [2]. Thus, a 12-min multiple-bed-position study can be performed in a single-bed position in under 30 s with comparable image quality, and the entire scan only taking 5 min. Imaging faster has a variety of benefits, such as reduction in respiratory motion, elimination of the need for sedation in certain patient groups, and enhanced patient throughput per unit of time in a single workday. In addition, imaging young patients or patients with pain or claustrophobia becomes vastly easier and more comfortable [4].

### 2.3. Ultrafast Acquisition for Motion Correction

Another benefit of total-body scanning involves the shorter acquisition time, thus resulting in less movement-induced blurring and allowing for ultrafast breath-hold equations to correct signal processing in moving regions. This is primarily due to this ultrafast tool’s ability to image in a single breath-hold, thus reducing respiratory motion artifacts and consequently improving target quantitation and localization on PET/CT imaging [5]. As a tangible example, total-body scanners may be able to generate a standardized uptake value (SUV)-based quantification of global lung function using ventilation/perfusion (V/Q) PET scanning with Gallium-68 (68Ga) radiolabeled tracers. Currently, the respiratory motion associated with this imaging technique makes determination of these volumes a challenging endeavor. Thus, the ultrafast acquisition associated with total-body scanning may be able to solve this issue. 

### 2.4. Detectability of Smaller Lesions

A major flaw associated with conventional fluorodeoxyglucose (FDG)-PET/CT technology involves its inability to detect metastatic deposits smaller than 5 mm due to its limited sensitivity. Total-body scanners, on the other hand, with ultrahigh sensitivity, good spatial resolution, and a long scan range, can detect small, low-density tumor deposits and micro-metastases. In addition, the high lesion SNR makes it possible to detect small lesions near the diaphragm affected by respiratory artifact. While improving the sensitivity and resolution can increase the detection of benign lesions as well, i.e., false positives, this novel technique may redefine the appearance of normal structures and provide clinicians with a wealth of information. 

### 2.5. Total-Body Dynamic Scanning

**Figure 1:**
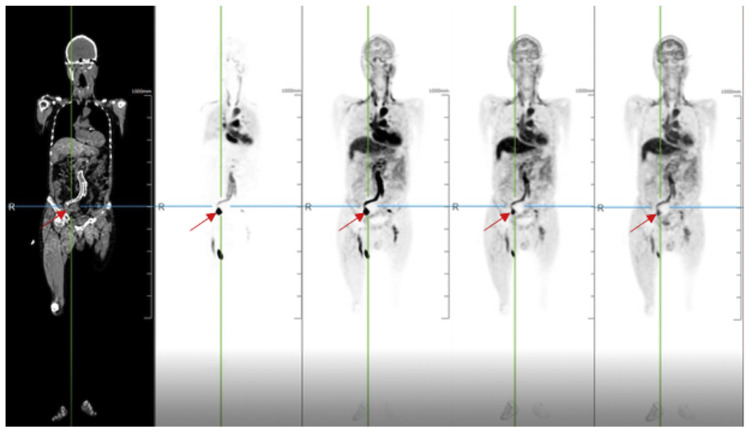
Radiotracer angiography. A 71-year-old man with a clipped aneurysm of an internal iliac artery underwent a total-body PET/CT scan. The dynamic imaging demonstrated that blood flow existed in the aneurysm at the arterial phase. Reprinted with permission from Ref. [7]. Copyright 2020 Elsevier.

In order to extract quantitative parameters from the temporal analysis of the radiotracer distribution in voxels, novel four-dimensional (4D) dynamic whole-body PET acquisition methods have been suggested [6,7] (Figure 1). After extracting the plasma input function from the acquired images, this method utilizes a Patlak-based modeling approach to provide an estimation of kinetic parameters such as the tracer uptake rate K_i_ (slope) [8]. Certain authors have recognized the superiority of this 4D dynamic over the traditional three-dimensional (3D) approach in tumor characterization and distinguishing inflammation versus malignancy [9]. However, the early studies evaluated this dynamic PET methodology using a one-step conventional system (15–20 cm), which limited its ability to assess multiorgan diseases (cancer and beyond). Thus, multistep dynamic PET acquisition protocols have been tested and note various strengths and limitations. For instance, this technique allows for whole-body K_i_-based analysis over a short time frame (whole-body (WB) imaging in 5–10 min). Ultimately, novel total-body PET prototypes have been successfully developed, with increased quality of 4D quantification and excellent temporal sampling (WB in 10–20 s), supporting its use in various clinical conditions. 

By extending the scan range to cover the entire body in one bed position and acquire dynamic projections of the entire body simultaneously, the total-body scanner provides the chance for accurate tracer kinetic analysis to study human physiology and biochemistry. The high SNR improves the quality of images, which aids in lesion detection and quantification [10]. Shi et al. have reported that total-body dynamic PET imaging with ultra-low activity (0.37 MBq/kg) provided equal performance to full-activity (3.7 MBq/kg) PET imaging when investigating the kinetic metrics of FDG in 20 human subjects [11]. With comparable image quality and kinetic analysis, the total-body scanner provides a variety of advantages with ultra-low-activity FDG, such as a smaller median effective radiation dose (owing to a lower dose of FDG). Moreover, it provides a 10-fold reduction in the file size of the raw PET data, and faster data processing, reconstruction, and transport [12].

### 2.6. Longer Acquisition Delay

Another benefit of total-body PET scanners is that the study can be obtained at much later time points after tracer injection, thus enhancing the contrast between the tumoral lesion and background tissue. Certain authors, such as Price et al., even note an increase in contrast between tumor and background tissue of approximately 4-fold if the interval between administration of FDG is increased [13]. This can be explained by a wider distribution of radiotracer on delayed images, greater accumulation in the tumor, renal excretion, and a greater washout from normal tissue [14]. Thus, images obtained with a longer delay may reveal additional information about the extent of disease and enable visualization of smaller or less tracer-avid lesions not seen on prior imaging [15]. In a series of total-body PET images conducted on rhesus monkeys, Berg et al. described excellent image quality even 30 days after tracer injection [16]. While most conventional scanners are unable to extend PET studies beyond 7–10 days, the high sensitivity and longer acquisition delay of total-body PET have the ability to revolutionize the field as we know it.

### 2.7. Detection of Distant Metastases and Vascular Complications

Whereas conventional whole-body PET/CT might miss distant metastases due to its limited scan range (that is, head to thigh), total-body PET can cover the entire body (head to toe) in a single bed position. While distal metastases in the lower extremities are oftentimes rare in most adult-onset malignancies, due to the lack of red bone marrow in an adult’s lower extremities, this technique may be beneficial in the pediatric population. In addition, this methodology can be utilized for those cancers with a higher probability of developing distant metastases in the extremities (such as renal cancer or sarcoma). 

Vascular complications are oftentimes observed within cancer patients as well, with a high incidence of both deep vein thrombosis (DVT) and pulmonary embolism in this population [2]. As whole-body PET/CT fails to image the lower extremities, common sites of venous thrombosis, these clots often go undetected. Thus, delayed total-body scanning in conjunction with FDG-PET/CT may revolutionize the management of cancer patients with DVT by identifying these clots earlier and with greater sensitivity [2]. 

## 3. Multiorgan Diseases

Now that the advantages of total-body PET scanning have been discussed, we will delve into the potential applications of this technique in a variety of clinical scenarios. 

### 3.1. Oncologic Applications

**Figure 2:**
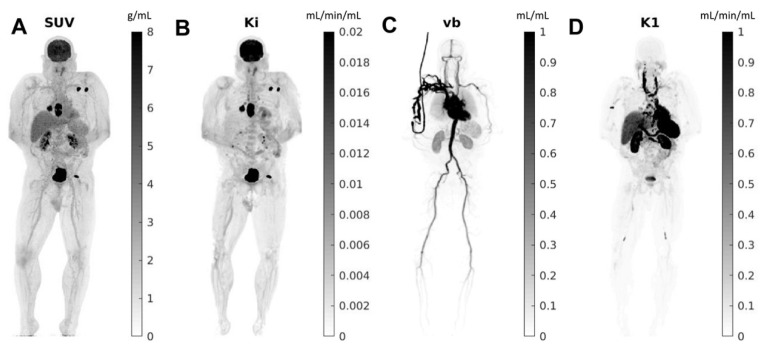
Total-body parametric images estimated from a 60-min dynamic ^18^F-FDG scan of a patient with metastatic cancer on the uEXPLORER: (**A**) SUV; (**B**) FDG net influx rate (Ki); (**C**) fractional blood volume (vb); and (**D**) FDG delivery rate (K_1_). Reprinted with permission from Ref. [19]. Copyright 2021 Elsevier.

As described earlier, the high sensitivity and dynamic range of total-body PET imaging allow for imaging at much later time points post radiotracer injection (up to 5–6 half times). This is particularly important as tumor contrast typically increases with time, given the clearance of tracer from other tissues. For example, most malignant lesions, including primary cancers and metastatic lesions, would show higher FDG uptake 2 h post-injection rather than 1 h. Thus, delayed imaging can add vital information regarding disease extent by enhancing tumor uptake and detecting smaller or lesser-avid lesions [17,18]. Total-body PET also affords the opportunity to obtain kinetic information using time series scans and kinetic modeling methods (Figure 2) [19]. This kinetic information can then be applied to identify small regions of tumoral infiltration, as some small lesions are below the resolution limit of the scanner and cannot be visualized, but sufficiently alter the kinetics within a voxel [10]. Such a paradigm allows for the delineation of low-grade diseases (cancers, inflammation/infection).

**Figure 3:**
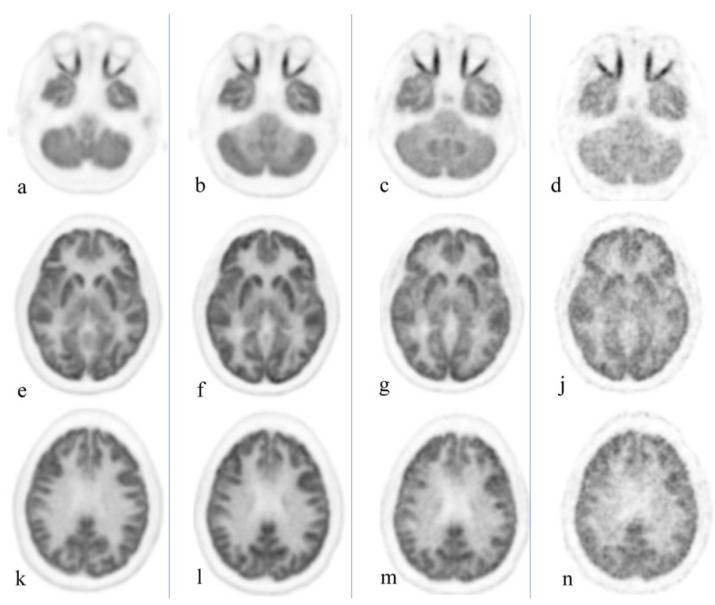
Healthy volunteer, total body PET/CT 20-min acquisition at 40 min, 3 h, 6 h, and 9 h after administration of 10 mCi of 18F-FGD. Cuts were selected at the orbit level (**a**–**d**), basal ganglia (**e**–**j**), and upper aspect of the ventricles (**k**–**n**). Brain uptake slowly decreases over time; the increase in noise level starts to become clinically evident at 9 h. Reprinted with permission from Ref. [4]. Copyright 2020 Elsevier.

Delayed imaging further differentiates malignancy and inflammatory lesions even in FDG-avid organs such as the brain, as inflammatory changes are often characterized by early wash out [4]. Figure 3 exemplifies the substantially reduced activity associated with delayed imaging of the brain, thus ultimately enhancing detection of small/recurrent brain tumors. This differentiation between malignancy and infection/inflammation can also be achieved utilizing dual time point imaging as indicated by Zhuang et al. [20]. Moreover, this technique may potentially increase FDG sensitivity for tumor detection, thus providing the geographic tumor distribution and quantification of microscopic deposits throughout the body, information vital for ultra-staging and management decisions [21]. 

### 3.2. Non-Oncologic Applications

**Figure 4:**
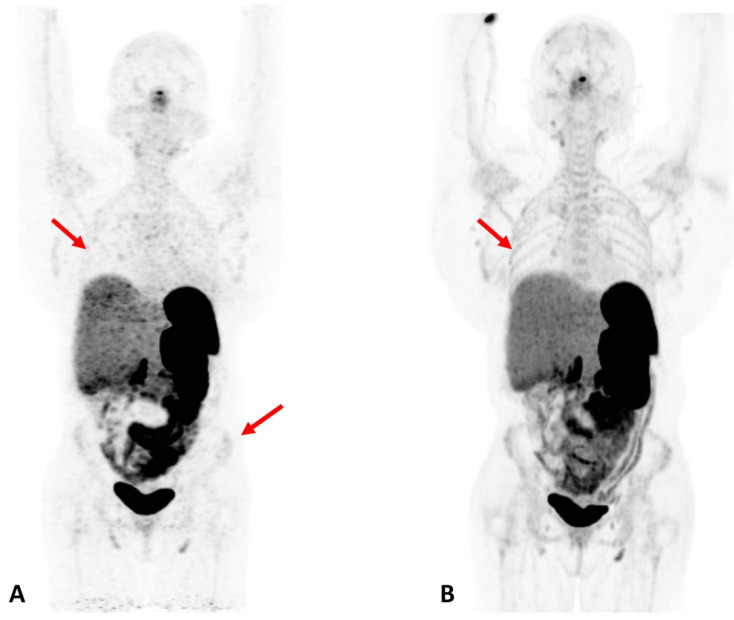
Patient with Cushing Syndrome post bilateral adrenalectomy. A dose of 167 MBq of 68Ga DOTATATE was injected, and a 20-min scan was performed after 60 min of uptake. (**A**) Baseline scan obtained on a conventional PET/CT scanner; (**B**) 6-month follow-up scan obtained on a total-body scanner. In the total-body PET/CT image, background noise is lower and the signal higher, allowing for clearer visualization of the liver. In addition, the increased signal level on the total-body scanner results in new or better visualization of bone details (arrows). Reprinted with permission from Ref. [23]. Copyright 2020 Elsevier.

Obesity and metabolic syndromes: Total-body PET imaging coupled with various metabolic tracers and advanced modeling techniques has great potential in the field of metabolic disorders. These techniques can enable both clinicians and researchers to investigate the pathophysiology of obesity-related metabolic disorders, and therefore contribute to the development of targeted interventions [22]. In addition, imaging multiple organs simultaneously can shed light upon the role of different organs and tissues (white and brown fat tissue, skeletal muscles, lower gastrointestinal (GI) tract, central nervous system (CNS), etc.) in these diseases. Such metabolic information can help elucidate targeted strategies for the diagnosis and treatment of metabolic disorders. Figure 4 exemplifies the enhanced organ/bone visualization associated with total-body PET scanning [23].

Musculoskeletal diseases (MSDs): As musculoskeletal diseases can occur anywhere throughout the body, total-body PET/CT is uniquely suitable for the detection and monitoring of these disorders, particularly degenerative or autoimmune arthritis, metabolic bone disease, muscle disorders (sarcopenia), and neoplasms [24]. The long axial coverage, high sensitivity, and excellent spatial resolution of total-body PET/CT will allow clinicians to assess total disease burden and guide clinical decision-making. Moreover, the lower radiation dose and faster acquisition associated with total-body PET/CT may enable this technique to play a critical role in both the pediatric population and the longitudinal monitoring of common disorders. 

Inflammatory and infectious conditions: Another unique characteristic of ultrasensitive total-body PET imaging involves its dynamic quantification of radiotracer uptake over time, providing noninvasive methods to study inflammatory and infectious diseases. The high signal-to-noise ratio can visualize disease reservoirs with a low pathogen burden, such as those seen in chronic disorders. By improving molecular imaging of cellular targets of viral infection (such as Angiotensin-converting enzyme 2 (ACE2) receptors in coronavirus 2019 (COVID-19) or cluster of differentiation 4 (CD4) cells in human immunodeficiency virus (HIV) patients) [25], total-body PET imaging may play a vital role in detecting organ involvement and elucidating disease pathogenesis. In addition, the decreased radiation dose can allow for longitudinal studies to assess trends in the immune response and downstream inflammatory consequences throughout the entire body. Furthermore, PET imaging of radiolabeled drugs can map the tissue-wide biodistribution and kinetics of various anti-infective agents. Lastly, this imaging technique can serially assess cell-based therapies in the setting of infections.

Cardiovascular diseases: With respect to the cardiovascular system, total-body dynamic PET imaging can provide invaluable data regarding ischemic, inflammatory, malignant, and infectious cardiac disorders. For example, this imaging technique can generate SUV time–activity curves for organs affected by systemic cardiovascular diseases such as sarcoidosis, amyloidosis, and vasculitis [26]. Moreover, total-body PET imaging can comparatively quantify regional blood flow in different organs simultaneously, which can then be adapted in the evaluation of malignant and ischemic disorders. In addition, this technique can be especially useful post-myocardial infarction (MI) as it can provide a comprehensive kinetic analysis of the ischemic effects on both cardiac and extracardiac tissue. This novel method will enable researchers to concurrently assess perfusion time–uptake defects in all vascular beds and map the physiologic perfusion fluctuations in organs at rest and during stress. Thus, this unique tool can significantly improve our understanding of the systemic effects and interactions of multiple cardiovascular disorders.

Total-body PET imaging also has great value in monitoring and detecting atherosclerotic disease, as current imaging techniques have a variety of limitations [2]. For instance, traditional structural imaging techniques are of limited utility in the prediction of plaque rupture. Standard PET techniques generate images with a suboptimal number of counts and thus a greater amount of noise [2]. Total-body scanning, on the other hand, can decrease the administered dose, delay acquisition, and reduce the scan time. This is turn would allow for serial monitoring in this patient population while still minimizing radiation exposure [2].

## 4. Miscellaneous Applications of Total-Body PET in Clinical Practice

### Differentiating between Residual Disease and Post-Therapy Changes

A major flaw associated with traditional anatomic imaging techniques such as CT scans or magnetic resonance imaging (MRI) is that these methods cannot reliably distinguish residual disease and post-therapeutic sequalae (such as post-surgical or post-radiation changes), thus generating false-positive cases. While conventional FDG-PET/CT holds a powerful diagnostic ability in this arena, a 3-month interval is still required post-therapy to minimize the likelihood of false-positive results. In addition, while the negative predictive value of FDG-PET/CT is excellent (~100%), its positive predictive value remains insensitive due to uncertainty between minimal residue and local inflammation. Recently, certain authors have suggested that an optimized dynamic signal analysis with total-body PET can differentiate minimal residual disease and post-therapeutic local inflammation, as kinetic parameters extracted from dynamic PET acquisition vary significantly in malignant and inflammatory processes.

Another flaw inherent in traditional FDG-PET/CT studies involves its inability to differentiate pseudo-progression and confirmed progression. This in turn can be misleading for clinicians analyzing response to treatment with systemic immunotherapy. To minimize these discrepancies, certain authors suggest delaying therapy assessment with FDG-PET/CT by several weeks after systemic therapies. However, this imaging delay may engender a loss of valuable time in both diagnosis and early treatment of progressive diseases. Total-body PET scanning can be invaluable in this regard, as it is able to differentiate progressive and pseudo-progressive disease both earlier and with higher sensitivity. This in turn can allow clinicians to initiate optimal therapies soon and ultimately improve patient care. However, further studies are necessary to determine the exact ability of total-body PET scanning in differentiating progressive and pseudo-progressive disease. 

## 5. Drug Development

Other authors have identified a promising niche for total-body PET/CT imaging in drug development. Currently, many laboratories utilize animal models to conduct pharmacokinetic and pharmacodynamic studies, but these models are fraught with limitations. In order to combat this, micro-dosing methods have been developed using highly sensitive imaging tools (such as single-photon emission computed tomography (SPECT) and PET) to introduce novel therapeutic agents to humans [27,28]. Micro-dosing ultimately facilitates drug development by initiating human involvement prior to phase I trials, thus hastening the decision-making process by quickly removing ineffective compounds from the drug pipeline [29].

In order to conduct these trials, very low doses of a radionucleotide-labeled compound are administered to determine plasma pharmacokinetics. The use of such sub-pharmacological doses in phase 0/micro-dose studies requires sensitive analytic tools such as PET to determine the drug’s effects. As total-body PET scanning allows the entire body to be imaged simultaneously, changes in a drug’s distribution throughout the entire body can be tracked, providing an understanding of how an agent can concentrate in tissues over time. Thus, clinicians can assess the pharmacokinetics and pharmacodynamics of novel therapeutic agents in all body organs, yielding vital information prior to launching expensive clinical trials [3,30]. In addition, utilization of these ultra-sensitive devices can provoke the development of novel imaging radiotracers, with only a low dose of agent necessary to establish pharmacokinetics and human dosimetry. Total-body PET can also measure slower kinetics relative to the half-life of a given radionucleotide across the entire body, improving the precision with which the cumulated activity and thus the absorbed dose is estimated.

In addition, a total-body pharmacokinetic assessment can allow for toxicologic studies of different agents. Pharmacokinetic studies in conjunction with total-body PET can be applied to determine the biological effects of various compounds and/or their destructive effects on the body.

## 6. Monitoring Cellular and Nanoparticle-Based Therapies

Recently, novel therapeutic strategies such as cell-based therapies (e.g., adoptive immunotherapy and stem-cell therapy) have attracted substantial attention in the field of oncology. To facilitate the development of these nanoparticle-mediated therapies, a reliable assessment tool with high sensitivity such as total-body PET technology is necessary to determine the in vivo distribution and biological fate of injected substances [31].

To this aim, labeling cells with long-lived radionucleotides prior to injection has been standard practice in the realm of nuclear medicine. After cell labeling, various noninvasive imaging modalities are applied to visualize these cells or nanoparticles in vivo, as well as monitor and quantify cell accumulation and function. While most cell-labeling methods utilize PET technology due to the high resolution and precise quantification of this technique, currently available PET scanners can only detect large cell/nanoparticle quantities and follow them relatively briefly. Total-body PET systems, on the other hand, can detect decreased quantities and follow them for extended periods post-injection. In addition, utilization of long-lived positron emitters, such as Zirconium-89 (half-life: 3.3 d), allows total-body PET devices to monitor the labeled nanoparticles in vivo for weeks or even a month [32,33]. Thus, total-body PET scanning is promising in measuring and enhancing the efficiency of cell-based therapies, as well as providing information on the delivery and retention of nanoparticles over extended time frames. These cell-tracking methods also offer a particular opportunity to image immune cell trafficking, which may elucidate the mechanism behind the variable outcomes inherent in cancer immunotherapy [34].

## 7. Multi-Tracer PET Studies (Cocktail Injection)

While FDG-PET/CT technology plays a pivotal role in imaging certain cancers, several malignant lesions cannot be detected using FDG due to the variable rates of glucose metabolism present in some cancers. To overcome this limitation, the novel strategy of cocktail injection has been developed, in which two radiopharmaceuticals are combined prior to a single PET acquisition [35]. For example, while FDG is a marker of glucose metabolism in lytic metastases, sodium fluoride (NaF) reflects the osteoblastic activity with a high potential for detecting blastic condensing metastases. Therefore, in the case of mixed metastatic presentations such as skeletal/marrow metastases in breast cancer, combining multiple tracers can improve the osteo-metastasis detection rate [36]. Similarly, other cocktail formulas have been suggested for different cancers, such as 68Ga-DOTATOC/FDG to image neuroendocrine tumors [37] or F-fluoroestradiol (FES)/FDG for hormone-dependent (estrogen receptor (ER)-positive) breast cancers [38]. 

Although cocktail therapy seems to play a promising role in the detection of malignancies, this technique has not been widely employed in routine clinical practice due to several limitations. For example, this technology cannot differentiate the signal from each tracer. However, certain authors, such as Karakatsanis et al., note that dynamic PET techniques could quantitatively extract both tracer kinetics and analyze them separately [21]. These authors also proposed a model for Patlak K_i_-driven analysis from dual-tracer FDG/NaF PET data, which may improve PET quantification and contrast in the target lesions. The application of such a “two in one” concept in total-body PET may improve tumor characterization by reducing radiation exposure and maximizing cost-effectiveness and patient comfort [39].

## 8. Personalized Medicine

Over the past decade, traditional medicine has been rapidly shifting to the concept of personalized medicine, including molecular targeted therapy, immunotherapy, and theranostics. These personalized medicine techniques have been highly successful in the field of clinical oncology, where clinicians can evaluate specific tumor markers and select patients who might benefit from a specific molecular-targeted therapy, thus maximizing the therapeutic effect and minimizing toxicity [40]. Consequently, certain advanced, automated software such as radiomics has been developed to extract more data from image-based features [41]. Radiomics is defined as the high-throughput extraction of quantitative features from radiographic images to describe tumor phenotypes objectively and quantitatively. The emerging field of radiomic analysis holds great promise in quantitative imaging, primarily because it can evaluate intra-tumoral heterogenicity. Such a quantitative analysis has recently attracted considerable attention due to its potential ability to identify cancer genetics and predict therapy outcome on an individualized basis.

In addition to highlighting imaging biomarkers related to intratumor heterogeneity, PET radiomics may also be useful in novel therapy paradigms such as immunotherapy, somatostatin receptor (SSTR) therapy, or prostate-specific membrane antigen (PSMA)-targeted theranostics. The potential usefulness of PET radiomics for personalized medicine has been widely reported in various cancers [42,43,44], as it allows for tumor marker evaluation, response prediction, and prognostication. For example, in non-small cell lung cancer, intra-tumoral heterogeneity, assessed by pretreatment PET, predicts patient survival and response to epidermal growth factor receptor (EGFR) tyrosine kinase inhibitors [45]. PET radiomics has also been suggested in immunotherapy, as tumor glucose metabolism is closely related to the immune landscape in the tumor microenvironment [46]. Hence, the textural analysis (radiomic) in PET allows for assessing the tumoral heterogenicity and subsequently opens opportunities for personalized medicine adapted for each patient.

Nevertheless, despite the promising clinical potential of radiomic analysis, several challenges need to be addressed when designing such studies. First, the quality of radiomic analysis is noise-dependent, and thus is closely related to the intrinsic system performance (partial volume effect). In addition, PET textural indices remain limited for small lesions, such as small-sized incidentalomas found in the thyroid or adrenal glands [47]. Thus, many studies exclude tumors with volumes <3–5 cm from radiomic analysis [48]. Another limitation of this analysis technique is respiratory motion, particularly in lung cancer patients [49]. Total-body PET imaging could address both limitations, as this ultrasensitive device enables reconstruction with a noise-limited high-resolution matrix, ultimately improving radiomic analysis of both small and moving lesions.

## 9. Summary

Given its ultrahigh sensitivity, excellent temporal resolution, extended scan range, and the ability to provide dynamic total body imaging, total-body PET technology offers a variety of opportunities to address currently unsolved clinical issues. In addition, the decreased injected activity and shorter acquisition duration of this technique increase its utility in certain patient populations. Small or low-FDG-avid tumor deposits can also be detected utilizing total-body PET scanning, due to the enhanced signal-to-noise ratio, high sensitivity, and capability for delayed imaging. Furthermore, this technique can also adequately image regions affected by respiratory motion. Total-body PET scanning also provides dynamic quantitative analysis from head to toe, presenting great prospects in clinical practice, such as differentiating inflammation from tumoral lesions, drug development and toxicology, monitoring cell-based therapies, and personalized medicine. While total-body scanners are only available in a few medical institutions, with their continued optimization, it is expected that they will be widely available in the future, offering new perspectives and a plethora of information. Although this modality is more expensive than conventional PET/CT techniques, the lower radiation exposure associated with total-body PET scanning allows physicians to repeat scans multiple times for the same radiation cost of one traditional scan. Nevertheless, both systematic and sophisticated studies are still needed to fully elucidate the strengths and weaknesses of total-body PET scanning.

## Figures and Tables

**Table 1:** Overview on existing TB PET/CT commercial systems. Table reproduced with permission from [1]. Copyright 2022 Springer Nature Switzerland AG.

System	Biograph Vision Quadra	Uexplorer	PennPET Explorer
Company/Facility	Siemens Healthineers	UC Davis and United Imaging Healthcare	UPenn, KAGE Medical, and Philips
Purpose	Clinical (human)	Clinical (human)	Clinical (human)
Bore diameter (cm)	78	76	n.a.
aFOV (cm)	106	194	64 (140 planned)
tFOV (cm)	n.a.	68.6	57.6
Photo-sensors	Analog SiPMs	Analog SiPMs (SensL)	Digital SiPMs (PDPC)
Scintillators	LSO (20 mm)	LYSO (18.1 mm)	LYSO (19 mm)
CTR (ps)	219	430	250
dE/E (%)	10.1	11.7	12.0
Spatial res. (mm)	3.4	2.9	4.0
Sensitivity (kcps/MBq)	174	174	55
NCER/Mcps (kBq/cc)	<2.5 (26)	<1.855 (9.6)	>0.001 (30)

## References

[1] Nadig V., Herrmann K., Mottaghy F.M., Schulz V. (2021). Hybrid total-body pet scanners—Current status and future perspectives. Eur. J. Nucl. Med. Mol. Imaging.

[2] Alavi A., Saboury B., Nardo L., Zhang V., Wang M., Li H., Raynor W.Y., Werner T.J., Høilund-Carlsen P.F., Revheim M.-E. (2022). Potential and Most Relevant Applications of Total Body PET/CT Imaging. Clin. Nucl. Med..

[3] Cherry S.R., Jones T., Karp J.S., Qi J., Moses W.W., Badawi R.D. (2018). Total-body PET: Maximizing sensitivity to create new opportunities for clinical research and patient care. J. Nucl. Med..

[4] Nardo L., Schmall J.P., Werner T.J., Malogolowkin M., Badawi R.D., Alavi A. (2020). Potential Roles of Total-Body PET/Computed Tomography in Pediatric Imaging. PET Clin..

[5] Nehmeh S.A., Erdi Y.E., Meirelles G.S.P., Squire O., Larson S.M., Humm J.L., Schöder H. (2007). Deep-inspiration breath-hold PET/CT of the thorax. J. Nucl. Med..

[6] Karakatsanis N.A., Lodge M.A., Tahari A.K., Zhou Y., Wahl R.L., Rahmim A. (2013). Dynamic whole-body PET parametric imaging: I. Concept, acquisition protocol optimization and clinical application. Phys. Med. Biol..

[7] Sui X., Liu G., Hu P., Chen S., Yu H., Wang Y., Shi H. (2021). Total-Body PET/Computed Tomography Highlights in Clinical Practice. PET Clin..

[8] Karakatsanis N.A., Zhou Y., Lodge M.A., Casey M.E., Wahl R.L., Zaidi H., Rahmim A. (2015). Generalized whole-body Patlak parametric imaging for enhanced quantification in clinical PET. Phys. Med. Biol..

[9] Rahmim A., Lodge M.A., Karakatsanis N., Panin V.Y., Zhou Y., McMillan A., Cho S., Zaidi H., Casey M.E., Wahl R.L. (2019). Dynamic whole-body PET imaging: Principles, potentials and applications. Eur. J. Nucl. Med. Mol. Imaging.

[10] Saboury B., Morris M.A., Farhadi F., Nikpanah M., Werner T.J., Jones E.C., Alavi A. (2020). Reinventing Molecular Imaging with Total-Body PET, Part I. PET Clin..

[11] Liu G., Hu P., Yu H., Tan H., Zhang Y., Yin H., Hu Y., Gu J., Shi H. (2021). Ultra-low-activity total-body dynamic PET imaging allows equal performance to full-activity PET imaging for investigating kinetic metrics of 18F-FDG in healthy volunteers. Eur. J. Nucl. Med. Mol. Imaging.

[12] Lan X., Fan K., Li K., Cai W. (2021). Dynamic PET imaging with ultra-low-activity of 18F-FDG: Unleashing the potential of total-body PET. Eur. J. Nucl. Med. Mol. Imaging.

[13] Price P.M., Badawi R.D., Cherry S.R., Jones T. (2014). Ultra Staging to Unmask the Prescribing of Adjuvant Therapy in Cancer Patients: The Future Opportunity to Image Micrometastases Using Total-Body 18F-FDG PET Scanning. J. Nucl. Med..

[14] Cheng G., Alavi A., Lim E., Werner T.J., Del Bello C.V., Akers S.R. (2013). Dynamic Changes of FDG Uptake and Clearance in Normal Tissues. Mol. Imaging Biol..

[15] Basu S., Kung J., Houseni M., Zhuang H., Tidmarsh G.F., Alavi A. (2009). Temporal profile of fluorodeoxyglucose uptake in malignant lesions and normal organs over extended time periods in patients with lung carcinoma: Implications for its utilization in assessing malignant lesions. Q. J. Nucl. Med. Mol. Imaging.

[16] Berg E., Gill H., Marik J., Ogasawara A., Williams S., Van Dongen G., Vugts D., Cherry S.R., Tarantal A.F. (2019). Total-Body PET and Highly Stable Chelators Together Enable Meaningful 89Zr-Antibody PET Studies up to 30 Days After Injection. J. Nucl. Med..

[17] Lodge M.A., Lucas J.D., Marsden P.K., Cronin B.F., O’Doherty M.J., Smith M.A. (1999). A PET study of 18 FDG uptake in soft tissue masses. Eur. J. Nucl. Med..

[18] Kubota K., Itoh M., Ozaki K., Ono S., Tashiro M., Yamaguchi K., Akaizawa T., Yamada K., Fukuda H. (2001). Advantage of delayed whole-body FDG-PET imaging for tumour detection. Eur. J. Nucl. Med..

[19] Wang Y., Li E., Cherry S.R., Wang G. (2021). Total-Body PET Kinetic Modeling and Potential Opportunities Using Deep Learning. PET Clin..

[20] Zhuang H., Pourdehnad M., Lambright E.S., Yamamoto A.J., Lanuti M., Li P., Mozley P.D., Rossman M.D., Albelda S.M., Alavi A. (2001). Dual Time Point 18F-FDG PET Imaging for Differentiating Malignant from Inflammatory Processes. J. Nucl. Med..

[21] Saboury B., Morris M.A., Nikpanah M., Werner T.J., Jones E.C., Alavi A. (2020). Reinventing Molecular Imaging with Total-Body PET, Part II: Clinical Applications. PET Clin..

[22] Chondronikola M., Sarkar S. (2021). Total-body PET Imaging: A New Frontier for the Assessment of Metabolic Disease and Obesity. PET Clin..

[23] Nardo L., Abdelhafez Y.G., Spencer B.A., Badawi R.D. (2021). Clinical Implementation of Total-Body PET/CT at University of California, Davis. PET Clin..

[24] Chaudhari A.J., Raynor W.Y., Gholamrezanezhad A., Werner T.J., Rajapakse C.S., Alavi A. (2021). Total-Body PET Imaging of Musculoskeletal Disorders. PET Clin..

[25] Henrich T.J., Jones T., Beckford-Vera D., Price P.M., VanBrocklin H.F. (2021). Total-Body PET Imaging in Infectious Diseases. PET Clin..

[26] Rodriguez J.A., Selvaraj S., Bravo P.E. (2021). Potential Cardiovascular Applications of Total-body PET Imaging. PET Clin..

[27] Lappin G., Noveck R., Burt T. (2013). Microdosing and drug development: Past, present and future. Expert Opin. Drug Metab. Toxicol..

[28] Bergstrom M. (2017). The use of microdosing in the drug development of small organic and protein therapeutics. J. Nucl. Med..

[29] Jekunen A.P., Pauwels E.K.J., Kairemo K.J.A. (2010). Microdosing in early lead discovery. Bioanalysis.

[30] Burt T., John C.S., Ruckle J.L., Vuong L.T. (2017). Phase-0/microdosing studies using PET, AMS, and LC-MS/MS: A range of study methodologies and conduct considerations. Accelerating development of novel pharmaceuticals through safe testing in humans—A practical guide. Expert Opin Drug Deliv..

[31] Kircher M.F., Gambhir S.S., Grimm J. (2011). Noninvasive cell-tracking methods. Nat. Rev. Clin. Oncol..

[32] Charoenphun P., Meszaros L.K., Chuamsaamarkkee K., Sharif-Paghaleh E., Ballinger J.R., Ferris T.J., Went M., Mullen G.E.D., Blower P.J. (2015). [89Zr]Oxinate4 for long-term in vivo cell tracking by positron emission tomography. Eur. J. Nucl. Med. Mol. Imaging.

[33] Bansal A., Pandey M.K., Demirhan Y.E., Nesbitt J.J., Crespo-Diaz R.J., Terzic A., Behfar A., DeGrado T.R. (2015). Novel 89Zr cell labeling approach for PET-based cell trafficking studies. EJNMMI Res..

[34] Akins E.J., Dubey P. (2008). Noninvasive Imaging of Cell-Mediated Therapy for Treatment of Cancer. J. Nucl. Med..

[35] Iagaru A., Mittra E., Yaghoubi S.S., Dick D.W., Quon A., Goris M.L., Gambhir S.S. (2009). Novel Strategy for a Cocktail 18F-Fluoride and 18F-FDG PET/CT Scan for Evaluation of Malignancy: Results of the Pilot-Phase Study. J. Nucl. Med..

[36] Roop M.J., Singh B., Singh H., Watts A., Kohli P.S., Mittal B.R., Singh G. (2017). Incremental Value of Cocktail 18F-FDG and 18F-NaF PET/CT Over 18F-FDG PET/CT Alone for Characterization of Skeletal Metastases in Breast Cancer. Clin. Nucl. Med..

[37] Barrio M., Czernin J., Fanti S., Ambrosini V., Binse I., Du L., Eiber M., Herrmann K., Fendler W.P. (2017). The Impact of Somatostatin Receptor–Directed PET/CT on the Management of Patients with Neuroendocrine Tumor: A Systematic Review and Meta-Analysis. J. Nucl. Med..

[38] Evangelista L., Dieci M.V., Guarneri V., Conte P.F. (2016). 18F-Fluoroestradiol Positron Emission Tomography in Breast Cancer Patients: Systematic Review of the Literature & Meta-Analysis. Curr. Radiopharm..

[39] Abgral R., Bourhis D., Salaun P.-Y. (2021). Clinical perspectives for the use of total body PET/CT. Eur. J. Nucl. Med. Mol. Imaging.

[40] Ha S. (2019). Perspectives in Radiomics for Personalized Medicine and Theranostics. Nucl. Med. Mol. Imaging.

[41] Lambin P., Rios-Velazquez E., Leijenaar R., Carvalho S., van Stiphout R.G.P.M., Granton P., Zegers C.M.L., Gillies R., Boellard R., Dekker A. (2012). Radiomics: Extracting more information from medical images using advanced feature analysis. Eur. J. Cancer.

[42] Bang J.-I., Ha S., Kang S.-B., Lee K.-W., Lee H.S., Kim J.-S., Oh H.-K., Lee H.-Y., Kim S.E. (2015). Prediction of neoadjuvant radiation chemotherapy response and survival using pretreatment [18F]FDG PET/CT scans in locally advanced rectal cancer. Eur. J. Nucl. Med. Mol. Imaging..

[43] Yip S.S., Kim J., Coroller T.P., Parmar C., Velazquez E.R., Huynh E., Mak R.H., Aerts H.J.W.L. (2017). Associations Between Somatic Mutations and Metabolic Imaging Phenotypes in Non–Small Cell Lung Cancer. J. Nucl. Med..

[44] Ha S., Park S., Bang J.-L., Kim E.-K., Lee H.-Y. (2017). Metabolic Radiomics for Pretreatment 18F-FDG PET/CT to Characterize Locally Advanced Breast Cancer: Histopathologic Characteristics, Response to Neoadjuvant Chemotherapy, and Prognosis. Sci. Rep..

[45] Park S., Ha S., Lee S.-H., Paeng J.C., Keam B., Kim T.M., Kim D.-W., Heo D.S. (2018). Intratumoral heterogeneity characterized by pretreatment PET in non-small cell lung cancer patients predicts progression-free survival on EGFR tyrosine kinase inhibitor. PLoS ONE.

[46] Choi H., Na K.J. (2018). Integrative analysis of imaging and transcriptomic data of the immune landscape associated with tumor metabolism in lung adenocarcinoma: Clinical and prognostic implications. Theranostics.

[47] Thuillier P., Bourhis D., Schick U., Alavi Z., Guezennec C., Robin P., Kerlan V., Salaun P.-Y., Abgral R. (2021). Diagnostic value of PET textural indices for malignancy of FDG-avid adrenal lesions. Q. J. Nucl. Med. Mol. Imaging.

[48] Orlhac F., Soussan M., Maisonobe J.-A., Garcia C.A., Vanderlinden B., Buvat I. (2014). Tumor Texture Analysis in 18F-FDG PET: Relationships Between Texture Parameters, Histogram Indices, Standardized Uptake Values, Metabolic Volumes, and Total Lesion Glycolysis. J. Nucl. Med..

[49] Yip S., McCall K., Aristophanous M., Chen A.B., Aerts H.J.W.L., Berbeco R. (2014). Comparison of Texture Features Derived from Static and Respiratory-Gated PET Images in Non-Small Cell Lung Cancer. PLoS ONE.

